# The role of premorbid and psychosocial factors in the cognitive assessment of persons with and without HIV in a low‐income area in South Africa

**DOI:** 10.1002/alz70857_096678

**Published:** 2025-12-24

**Authors:** Anna Jane Dreyer, Celine Le Roux, Kevin G. F. Thomas, Caroline Sabin, Alan Winston, Saye Khoo, John Joska, Sam Nightingale

**Affiliations:** ^1^ University of Cambridge, Cambridge, United Kingdom; ^2^ University of Cape Town, Cape Town, South Africa; ^3^ University of Pretoria, Pretoria, South Africa; ^4^ University College London, London, United Kingdom; ^5^ Imperial College Healthcare NHS Trust, London, United Kingdom; ^6^ Imperial College London, London, United Kingdom; ^7^ University of Liverpool, Liverpool, United Kingdom

## Abstract

**Background:**

HIV can affect the brain leading to cognitive impairment. Accurate assessment of cognitive impairment requires consideration of the contribution of psychosocial and educational factors to cognitive test performance in people with and without HIV. Most HIV‐cognition studies only control for the standard demographic variables of age, sex, and years of education, which may have led to overestimation of cognitive impairment caused by HIV.

**Method:**

Participants (*N* = 273, 18‐55 years), 178 of which were living with HIV, were recruited from a low‐income area in Cape Town, South Africa. They were asked to complete a comprehensive cognitive assessment (7 domains) and a measure of premorbid cognitive performance (WAIS‐SA Information). We collected standard demographic variables typically controlled for HIV‐cognition studies (age, sex, years of education), and additional 12 psychosocial measures: 5 assessing current circumstances (income, occupation, assets, accommodation, depressive symptoms) and 7 assessing childhood circumstances (quality of education, primary caregiver occupation and highest level of education, household assets as child, exposure to childhood trauma and violence). Using linear regression models, we investigated the association between HIV status and global cognitive performance (global T‐score), and premorbid cognitive performance. We then investigated the association adjusting for standard demographic variables, and then after additional adjustments for psychosocial variables.

**Result:**

In unadjusted analysis, persons with HIV had 3.72 units lower global T (*p* < .001) and 1.35 points lower premorbid scores (*p* < .001) compared with persons without HIV. Persons with HIV had 0.5 fewer years of education (*p* < .001), and significantly lower scores in 8/12 psychosocial variables (*p*s < .017). Adjustment for demographic variables alone reduced the effect of HIV on global T by 26.9% to 2.72 (*p* < .001), and on premorbid scores by 28.9% to 0.96 (*p* = .001). Additional adjustment for psychosocial variables reduced the HIV effect on global T by 40.3% to 2.22 (*p* = .004), and on premorbid scores by 48.2% to 0.70 (*p* = .057).

**Conclusion:**

People with HIV have lower psychosocial indices than those without HIV, which is associated with lower premorbid cognitive performance and lower cognitive test scores. Adjustment for the standard demographic variables alone incompletely mitigates this. Failure to consider psychosocial factors will lead to overestimation of cognitive impairment caused by HIV.

